# Lactobacilli Inactivate *Chlamydia trachomatis* through Lactic Acid but Not H_2_O_2_

**DOI:** 10.1371/journal.pone.0107758

**Published:** 2014-09-12

**Authors:** Zheng Gong, Yesmin Luna, Ping Yu, Huizhou Fan

**Affiliations:** 1 Department of Pharmacology, Rutgers University Robert Wood Johnson Medical School, Piscataway, New Jersey, United States of America; 2 Department of Immunology, Central South University Xiangya Medical School, Changsha, Hunan, China; University of Manitoba, Canada

## Abstract

*Lactobacillus* species dominate the microbiome in the lower genital tract of most reproductive-age women. Producing lactic acid and H_2_O_2_, lactobacilli are believed to play an important role in prevention of colonization by and growth of pathogens. However, to date, there have been no reported studies characterizing how lactobacilli interact with *Chlamydia trachomatis*, a leading sexually transmitted bacterium. In this report, we demonstrate inactivation of *C. trachomatis* infectivity by culture media conditioned by *Lactobacillus crispatus, L. gasseri* and *L. jensenii*, known to be dominating organisms in the human vaginal microbiome. *Lactobacillus* still cultures produced lactic acid, leading to time- and concentration-dependent killing of *C. trachomatis.* Neutralization of the acidic media completely reversed chlamydia killing. Addition of lactic acid into *Lactobacillus-*unconditioned growth medium recapitulated the chlamydiacidal activity of conditioned media. The H_2_O_2_ concentrations in the still cultures were found to be comparable to those reported for the cervicovaginal fluid, but insufficient to inactivate chlamydiae. Aeration of *Lactobacillus* cultures by shaking markedly induced H_2_O_2_ production, but strongly inhibited *Lactobacillus* growth and lactic acid production, and thus severely affected acidification, leading to significantly reduced chlamydiacidal efficiency. These observations indicate lactobacilli inactivate chlamydiae primarily through maintaining acidity in a relatively hypoxic environment in the vaginal lumen with limited H_2_O_2_, which is consistent with the notion that women with higher vaginal pH are more prone to sexually transmitted *C. trachomatis* infection. In addition to lactic acid, formic acid and acetic acid also exhibited potent chlamydiacidal activities. Taken together, our findings imply that lowering the vaginal pH through engineering of the vaginal microbiome and other means will make women less susceptible to *C. trachomatis* infection.

## Introduction

In the US, more than half of infections reported to the Center for Disease Control are sexually transmitted infections (STI) [1], [2]. The number one sexually transmitted bacterial pathogen in the US is *Chlamydia trachomatis* [1], [2], a Gram-negative bacterium requiring eukaryotic cells as hosts for replication [3]. *C. trachomatis* STI is also highly prevalent in the rest of the world [4], [5], [6]. Initial *C. trachomatis* replication in the lower genital tract causes vaginitis and cervicitis [1], [2], [7]. As the pathogen disseminates upwards to the uterus and oviducts, endometritis and salpingitis occur, which may lead to abortion, premature birth and ectopic pregnancy [1], [2], [7], [8].

There are more than 10 genital *C. trachomatis* serovars [7]. Following acute infection, human hosts develop only short-lived, serovar-specific protective immunity [7]. Therefore, recurrent infection with either the same and/or different serovars is common. Since *C. trachomatis-*infected cases are often asymptomatic or exhibit very mild symptoms, only a small proportion of the infected women seek medical treatment [1], [2], [7]. Without proper antibiotic treatment, repeated infection-mediated inflammation leads to severe oviductal fibrosis, which constitutes the leading cause of tubal factor infertility [8], [9], [10], [11]. Therefore, although both men and women are susceptible to *C. trachomatis*, urogenital infection disproportionally affects the wellbeing of women.

The vaginal microbiome in most reproductive-age women is dominated by lactic acid-producing bacteria [12], [13]. High throughput DNA sequencing analyses confirmed that in the majority of these women in North America, the vaginal microbiome is dominated by *Lactobacillus crispatus, L. gasseri, L. iners* or *L. jensenii* [13]; and there is a correlation between taxa profiles and Nugent scores, which are used to diagnose bacterial vaginosis [13], [14], [15].

*Lactobacillus* species generates three principal types of antimicrobials, lactic acid, H_2_O_2_, and a large number of antimicrobial peptides [16], [17]. Because of high concentrations of lactic acid, the acidity in the vaginal lumen may become lower than pH 4 [12], [13], [18]. While a number of population-based studies suggest an important role of acidity in the health of women's genital tract [19], [20], [21], [22], [23], [24], [25], laboratory researchers seem to have mostly focused on H_2_O_2_ (eg, [26], [27], [28], [29], [30]); it has been generally assumed that H_2_O_2_ functions as an important pathogen deterrent there. However, some recent studies found that physiological concentrations of H_2_O_2_ in the cervicovaginal fluid are unable to protect against organisms associated with bacterial vaginosis [18], [31].

Bacterial vaginosis and high Nugent scores have been associated with increased risks of chlamydial STIs [20], [24], [25]. However, to the best of our knowledge, there have been no experimental documentation on if and how vaginal microbiome influences chlamydial pathogenicity. This lack of basic understanding, and a randomized, double-blind and placebo-controlled trial showing therapeutic value of lactobacilli for curing bacterial vaginosis [32] have prompted us to study how three major vaginal *Lactobacillus* species interact with *C. trachomatis* [13]. Our *in vitro* findings indicate that these H_2_O_2_-producing organisms inactivate the infectivity of *C. trachomatis* primarily through an acid-dependent mechanism, and in contrast, H_2_O_2_ plays an undetectable role in helping the human host defend against *C. trachomatis* under physiological conditions. Significantly, we report that induction of H_2_O_2_ production in these bacteria results in inhibition of lactic acid production and chlamydiacidal activity. Accordingly, we propose that for the prevention of chlamydial STI, efforts should be focused on colonization and maintenance of high lactic acid-producing lactobacilli in the female genital tract.

## Materials and Methods

### Materials

Dulbecco's modified Eagle's medium (DMEM) with high glucose (4.5 g/L) and 110 mg sodium pyruvate, fetal bovine serum (FBS), lactic acid (86% solution, equal ratio of L- and D-lactic acid), formic acid, paraformaldehyde, bovine liver catalase, bovine heart L-lactic dehydrogenase, *Lactobacillus leichmanii* D-lactic dehydrogenase, glycine, hydrazine and KMnO_4_ were purchased from Sigma Aldrich. H_2_O_2_ (3% solution; American Choice) was purchased from a local pharmacy (Walgreen). Anhydrous, acetone-free methanol and acetic acid glacial were products of J.T Baker. A Pierce Quantitative Peroxide Assay Kit was purchased from Fisher Scientific.

### Bacterial strains

A clone of GFP-L2, derived by transforming *C. trachomatis* serovar L2, a lymphogranuloma venereum (LGV) pathogen, with an expression plasmid for a green fluorescence protein (GFP-L2) [33], was obtained by limiting dilution. A GFP-expressing *C. trachomatis* serovar D clone designated GFP-CTD1 [34] was a generous gift from Dr. Guangming Zhong (University of Texas Health Sciences Center at San Antonio). The *C. trachomatis* strains were expanded using McCoy cells as the host. Their elementary bodies (EBs), the infectious form of the pathogen, were purified through RenoCal gradient centrifugation as described [35]. EB aliquots were stored in a −80°C freezer.

*L. crispatus* strains 33197 (Lc33197) and 33820 (Lc33820), *L. gasseri* strain 33323 (Lg33323), *L. jensenii* strain 25258 (Lj25258), and *Shigella flexneri* 2a strain 2457T (*S. flexneri* 2457T) were purchased from ATCC. Upon receipt, *Lactobacillus* strains were grown at 37°C in MRS Lactobacilli broth (Difco) as still cultures in a humidified 5% CO_2_ incubator, whereas *S. flexneri* 2457T was grown in Luria-Bertani (LB) broth in a 37°C water-bath shaker incubator. Aliquots of the first passage were frozen at −80°C after the addition of glycerol to a final concentration of 10%. For this study, the strains were recovered from the deep freezer and subcultured daily at a ratio of ∼1∶1,000. *Lactobacillus* cultures were discarded by 10 passages after recovery from one of the frozen passage 1 vials.

### Lactobacillus still culture

Routine still culture was performed using 14.5 ml Falcon plastic culture tubes, 50 ml plastic conical tubes or 150 ml glass bottles, and a 37°C, humidified 5% CO_2_ incubator except for experiments described below that compared shaken and still cultures. Containers were filled no more than half-full and caps were kept loose to allow for air exchange between inside and outside of the containers.

### *Lactobacillus* shaken culture

30 ml glass culture tubes each containing a 6 ml culture were placed on a TC-7 rolling drum shaker with a speed set at high in a 37°C incubator with neither artificial humidification nor CO_2_ supplementation. For experiments comparing the effects of shaking on H_2_O_2_ production and other metabolic activities, control still cultures were obtained with same culture tubes in the same incubator.

### Preparation of *Lactobacillus-*conditioned media (LCM)

Cultures were centrifuged at 3,000 rpm for 10 min in a Beckman GPR centrifuge. The supernatants, defined as LCM, were collected. pH values of the supernatants were measured using a Fisher pH meter, which was pre-calibrated with pH 4.0 and pH 7.0 standards. LCM were then sterilized by passing through 0.2 micron filters, and stored at 4°C or −20°C in air-tight tubes. In some experiments, a portion of the LCM was adjusted to pH 7.0 before filter-sterilization. The neutralization was performed first with a 10 M NaOH solution and then a 1 M NaOH solution when needed. For most experiments, LCM was used immediately or within 5 days after preparation. All LCM used in catalase experiments was prepared fresh prior to the experiments.

### Determination of *Lactobacillus* concentration

Bacteria concentrations were estimated by measuring optical density at 600 nm (OD_600_) using an Amersham spectrophotometer. When necessary, cultures were diluted with MRS until the reading fell below 0.7 where the OD_600_ remained linear for the bacterial concentration.

### H_2_O_2_ quantitation

Quantitation of H_2_O_2_ was performed with a Pierce Quantitative Peroxide Assay Kit following manufacturer's instruction. This kit detects H_2_O_2_ by measuring a purple product, which is produced from the reaction of xylenol orange with H_2_O_2_-derived Fe^3+^, and has an absorbance maximum at 560 nm. LCM was 1∶10 diluted with the assay buffer before it was added to the assay mix. Standard curves were established by substituting LCM with commercial H_2_O_2_ diluted with the MRS broth.

### Lactic acid assays

Lactic acid in LCM was measured by using lactic dehydrogenase, which converts the cofactor NAD to NADH, resulting in an increase in absorbance at 340 nm. Assays were performed using L- and D-lactic dehydrogenase separately in a 96-well format. Each assay contained 0.3 M glycine, 0.3 M hydrazine, 5 mM NAD, 2 µl LCM, and 0.1 unit of dehydrogenase (or equal volume of phosphate-buffered saline [PBS] containing 1% bovine serum albumin), in a total volume of 200 µl. The amount of lactic dehydrogenase added into the reactions had no effect on *A*_340_. Standard curves were established by substituting LCM with commercial lactic acid diluted in the MRS broth. The sum of L- and D-lactic acid is presented.

### *C. trachomatis* killing tests

On ice, 10 µl of EB suspension was diluted with 290 µl 0.9% NaCl. 10 µl of the diluted EB suspension was mixed with 100 µl of LCM (or MRS containing indicated concentrations of lactic acid) or control MRS. Initial experiments showed that treatment with MRS had no adverse effect on the viability of EBs, as compared to the cell culture medium DMEM supplemented with 10% FBS (data not shown); therefore, the DMEM control was omitted in later experiments. The mixes were incubated at room temperature for 1 h, and then subjected to 10 fold serial dilution with DMEM containing 10% FBS, 1 µg/ml cycloheximide and 10 µg/ml ampicillin. Serial dilution was performed on 96-well plates. The dilutions of 1∶100, 1∶1,000 and 1∶10,000 were transferred onto McCoy cell monolayers at 80–90% confluence on 96-well plates. The undiluted mix was not inoculated McCoy cells because the polysobate-80 in MRS and high acidity in some LCM were toxic to the host cells, which was evident by observation of unstained cultures and significantly decreased staining by the vital stain neutral red (data not shown). The dilution of 1∶10 was not inoculated either, because1∶10 diluted control MRS had a moderate inhibitory effect (∼2 fold decrease) on intracellular chlamydial growth (the inhibition of chlamydial growth by MRS became undetectable at 1∶40 or higher dilutions) (data not shown). For experiments with GFP-CTD1, plates were first subjected to centrifugation (3,000 rpm, Beckman GPR) to facilitate infection before they were placed in the incubator. 36 h postinoculation, plates were removed from the incubator and placed on ice. Subsequent fixation procedures were performed with the plates kept on ice and solutions stored at 4°C. Media were removed. 3.5% paraformaldehyde, prepared in PBS, was added to the monolayers. 15 min later, cells were washed 3 times with Tris-buffered saline (pH 8.0), treated with methanol for 10 min, washed twice with PBS, and kept in 100 µl PBS. Inclusions were enumerated using an Olympus IX51 fluorescence microscope [36].

### Catalase treatment

Lyophilized bovine liver catalase was reconstituted with deionized water to 10 mg/ml (20–50 units/µl). Aliquots were stored at −80°C. H_2_O_2_ removal reactions were carried out by adding 20 µl of the catalase preparation to 1.0 ml LCM or bacterial suspension (GFP-L2 EBs or *S. flexneri* 2457T) prepared with MRS supplemented with exogenous H_2_O_2_. Final protein concentration of the catalase was 0.2 mg/ml. Control reactions received an equal volume of 0.9% NaCl. After mixing and incubation at room temperature for 1 h, H_2_O_2_-depleted LCM were used to treat EBs, whereas GFP-L2 suspensions were inoculated onto McCoy cells and inclusion-forming units were determined as described above; *S. flexneri* suspensions were inoculated onto LB Agar plates following 10 fold serial dilutions, and colony-forming units were determined following overnight incubation at 37°C. To demonstrate the enzyme activity of catalase, 3% H_2_O_2_ was diluted 10 fold with 0.17 M lactate-NaOH (pH4.0). The resulting 0.3% H_2_O_2_ solution was treated with catalase or 0.9% NaCl as described above; remaining H_2_O_2_ was measured as described below.

### Catalase assay

Catalase activity was determined by measuring the amounts of KMnO_4_ needed to titrate H_2_O_2_ before and after catalase treatment. KMnO_4_ titration was performed at a mini-scale. Briefly, 2.5 ml H_2_O, 0.5 ml 3 M H_2_SO_4_ and 100 µl 0.3% H_2_O_2_ that had been treated with catalase or NaCl were sequentially added to a 50 ml glass beaker. While mixing, a Fisher plastic transfer pipette was used to add 20 mM KMnO_4_ to the beaker drop wise until a faint pink color persisted for 30 s. The amount of KMnO_4_ solution consumed was determined by weighing the pipette carrying the 20 mM KMnO_4_ solution before and after the titration.

### Statistical analysis

A two-sided *t* test, unless indicated otherwise, was performed on Microsoft Excel to analyze EB titers, H_2_O_2_ production, lactic acid production, pH values and *Lactobacillus* concentrations. A significant difference was defined as a *P* value of <0.05. Single and double asterisks in figures denote *P*<0.05 and *P*<0.01, respectively.

## Results

### LCM concentration- and time-dependent inactivation of GFP-L2

To deduce the effect of vaginal lactobacilli on chlamydial STI, we determined how LCM affects the viability of *C. trachomatis* EBs as detailed in “Materials and Methods”. Although *in vitro* studies examining antimicrobial effects of lactobacilli are typically done in co-culture systems, such a system cannot be used when the target microbe is an obligate intracellular organism because host cells cultured *in vitro* cannot tolerate the acidity produced by lactobacilli. We chose four strains of lactobacilli belonging to three species, *L. crispatus, L. gasseri* and *L. jensenii* for this study. These three species are common vaginal lactobacilli in North American women of reproductive ages, and are associated with various levels of vaginal acidity [13]. They acidify the MRS medium efficiently. Previous studies have shown they all produce H_2_O_2_ in MRS [37], [38], [39]. *L. iners* is another dominant *Lactobacillus* species in the human vagina, but it was not included in this study because it does not grow in MRS, and we found that this organism failed to acidify the NYC III medium and defibrinated sheep blood-supplemented trypticase soy broth, which support its growth to a very limited degree (data not shown).

**Figure 1:**
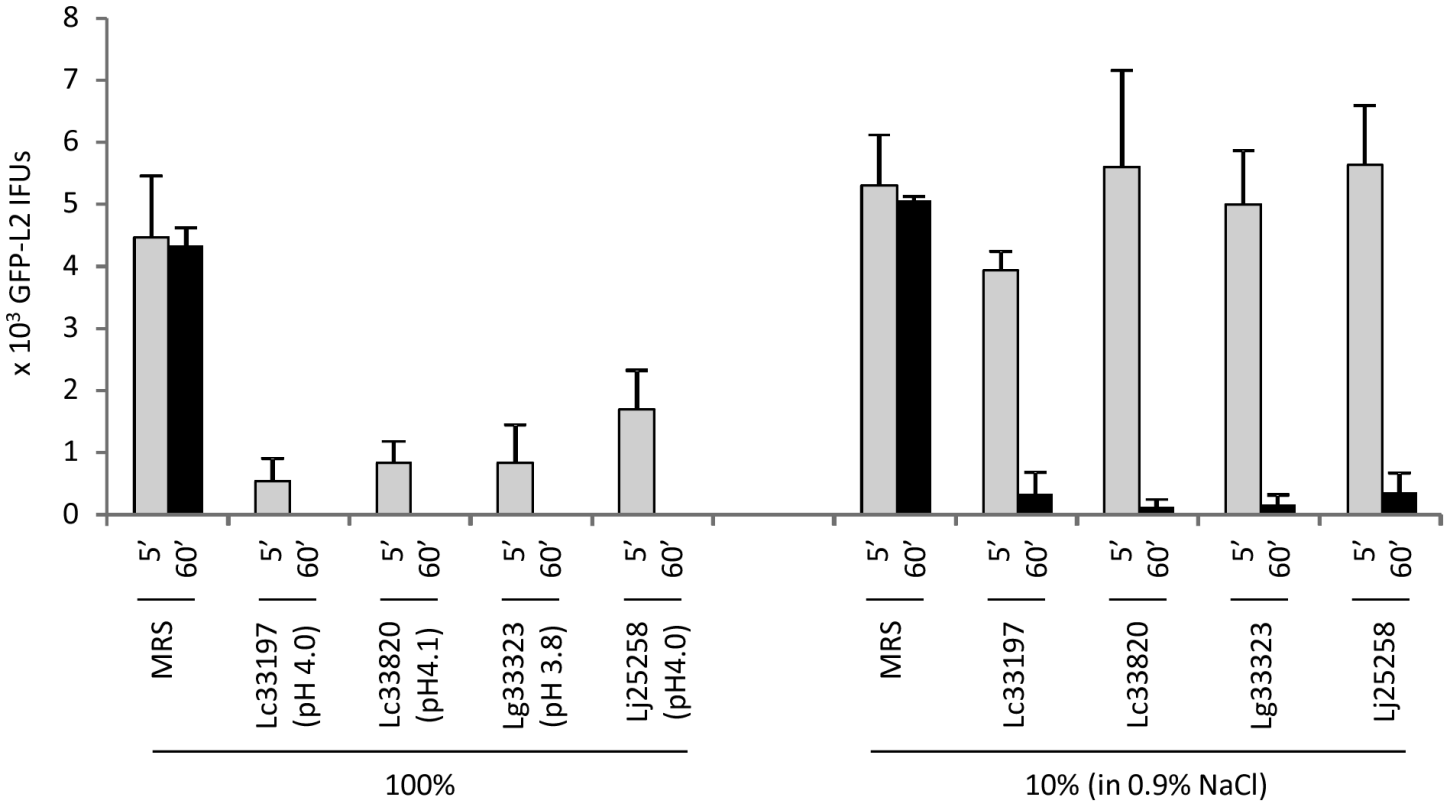
Time- and concentration-dependent inactivation of elementary bodies (EBs) of GFP-L2, derived from *C. trachomatis* serovar L2, by *Lactobacillus-*conditioned medium (LCM). LCM was collected from overnight still cultures of *L. crispatus* 33197 (Lc33197), *L. crispatus* 33820 (Lc33820), *L. gasseri* 33323 (Lg33323) and *L. jensenii* 25258 (Lj25258). pH values of undiluted (100%) LCM are shown. EBs were treated with undiluted LCM or LCM diluted 10 fold with 0.9% NaCl for 5 min or 1 h. Following treatments, surviving EBs were serially diluted and then inoculated onto McCoy monolayers; resulting inclusion-forming units were scored by fluorescence microscopy. Values were averages ± standard deviations of triplicate experiments.

We first determined the effects of LCM, harvested from overnight still cultures, on the viability of GFP-L2 EBs. Typically, LCM collected from these cultures had a pH near 4.0, which is within the range of acidity in healthy women with microbiome dominated by lactobacilli [12], [13], [18], as indicated in Fig. 1. Undiluted (100%) LCM killed the (vast) majority of GFP-L2 EBs after only 5 min treatment, and inactivated all the EBs after an hour. While 5 min treatment with 10% LCM, prepared by dilution with 0.9% NaCl, failed to show a chlamydiacidal effect, extension of treatment time to 1 h resulted in killing of more than 90% EBs. For remaining experiments of this report, EBs were treated with undiluted LCM or lactic acid-acidified MRS for 1 h.

### Progressively increased chlamydiacidal activities during the course of *Lactobacillus* growth

**Figure 2:**
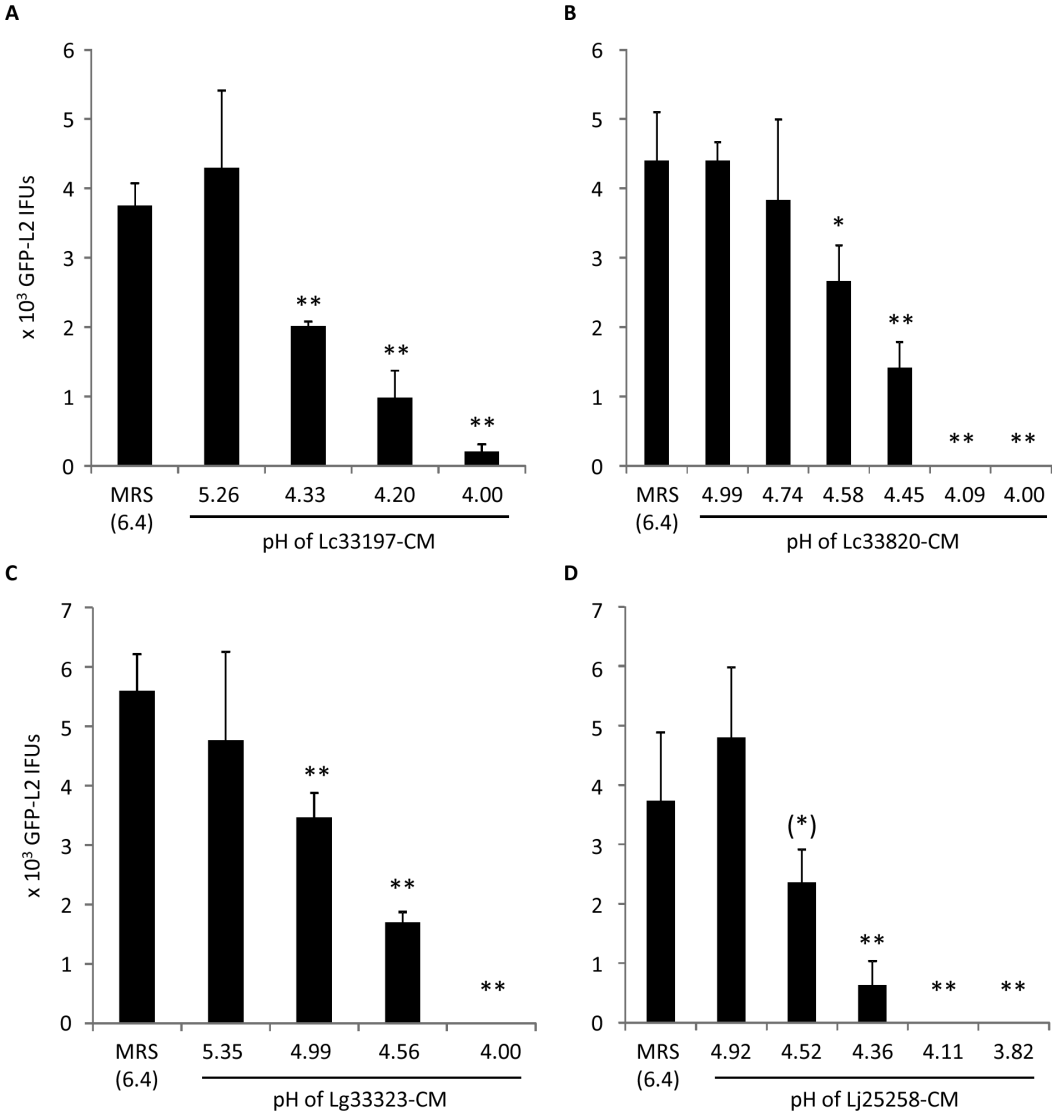
Effects of LCM longitudinally collected from still cultures of Lc33197 (A), Lc33820 (B), Lg33323 (C) and Lj25258 (D). pH values of LCM are shown on the horizontal axis. GFP-L2 EBs were treated for 1 h. Surviving bacteria were quantified as outlined in Fig. 1 legend. Values were averages ± standard deviations of triplicate experiments. Single and double asterisks above LCM-treated samples denote statistically decreased IFUs (P<0.05 and P<0.01, respectively) as compared to control MRS *Lactobacillus* medium-treated samples. The parenthetic asterisk indicates statistically decreased IFUs in samples treated with LCM (pH 4.52), as compared to IFUs that survived the treatment with LCM (pH 4.92), although the *P* value between control MRS (pH 6.4) and MRS (pH 4.52) was 0.068.

Lactic acid and H_2_O_2_ are considered two major antimicrobials from lactobacilli. Therefore, we performed a series of experiments to determine the roles of these two antimicrobials in EB inactivation. First, we collected LCM at different points following subculturing, and determined their effects on the viability of GFP-L2 EBs. The pH values of the LCM were recorded. Initially, we also attempted but failed to measure the concentrations of H_2_O_2_ with KMnO_4_ because the level of H_2_O_2_ was below the limit of the assay, which was further complicated by the interference of the assay with MRS (data not shown). As shown in Fig. 2, LCM from all the four *Lactobacillus* strains displayed a correlation between acidity and chlamydiacidal activity. Significant killing was undetectable until the pH was at or below 5; complete or near complete EB inactivation became apparent when the pH reached ∼4.0. Therefore, it appears that acidity of LCM is important for inactivation of EBs.

### Reversal of chlamydiacidal activities by increasing LCM pH

**Figure 3:**
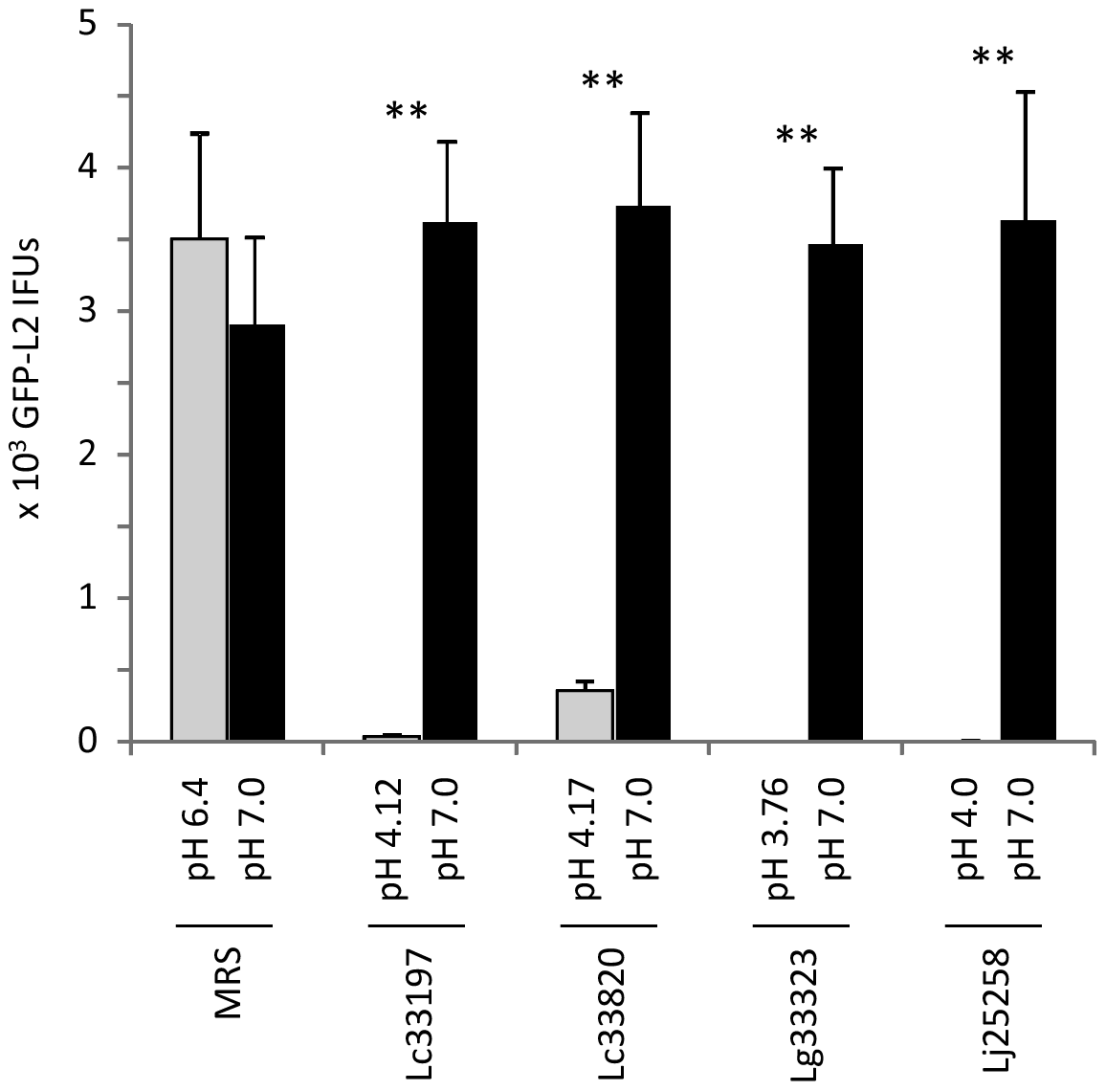
Complete reversal of chlamydiacidal activity with pH-neutralized LCM. LCM from overnight still cultures of *Lactobacillus* strains or control MRS broth was adjusted to pH 7.0. GFP-L2 EBs were treated with pH-unadjusted LCM (pH value shown for each on the horizontal X-axis) or neutralized LCM for 1 h. Surviving bacteria were quantified as outlined in Fig. 1 legend. Values were averages ± standard deviations of triplicate experiments. Double asterisks above LCM-treated samples denote highly statistically increased IFUs (P<0.01) as a result in treatment with neutralized LCM as compared to pH-unadjusted LCM.

To further determine if low pH is needed for the chlamydiacidal effect of LCM, we adjusted LCM from overnight still cultures with NaOH to pH 7, and compared their effects on the viability of GFP-L2 EBs with the effects of pH-unadjusted LCM. Without exception, the neutralization resulted in complete loss of chlamydiacidal activity (Fig. 3). These results suggest that low pH is absolutely required for the inactivation of EBs by LCM.

### Recapitulation of LCM-mediated chlamydiacidal effects with lactose-supplemented MRS

**Figure 4:**
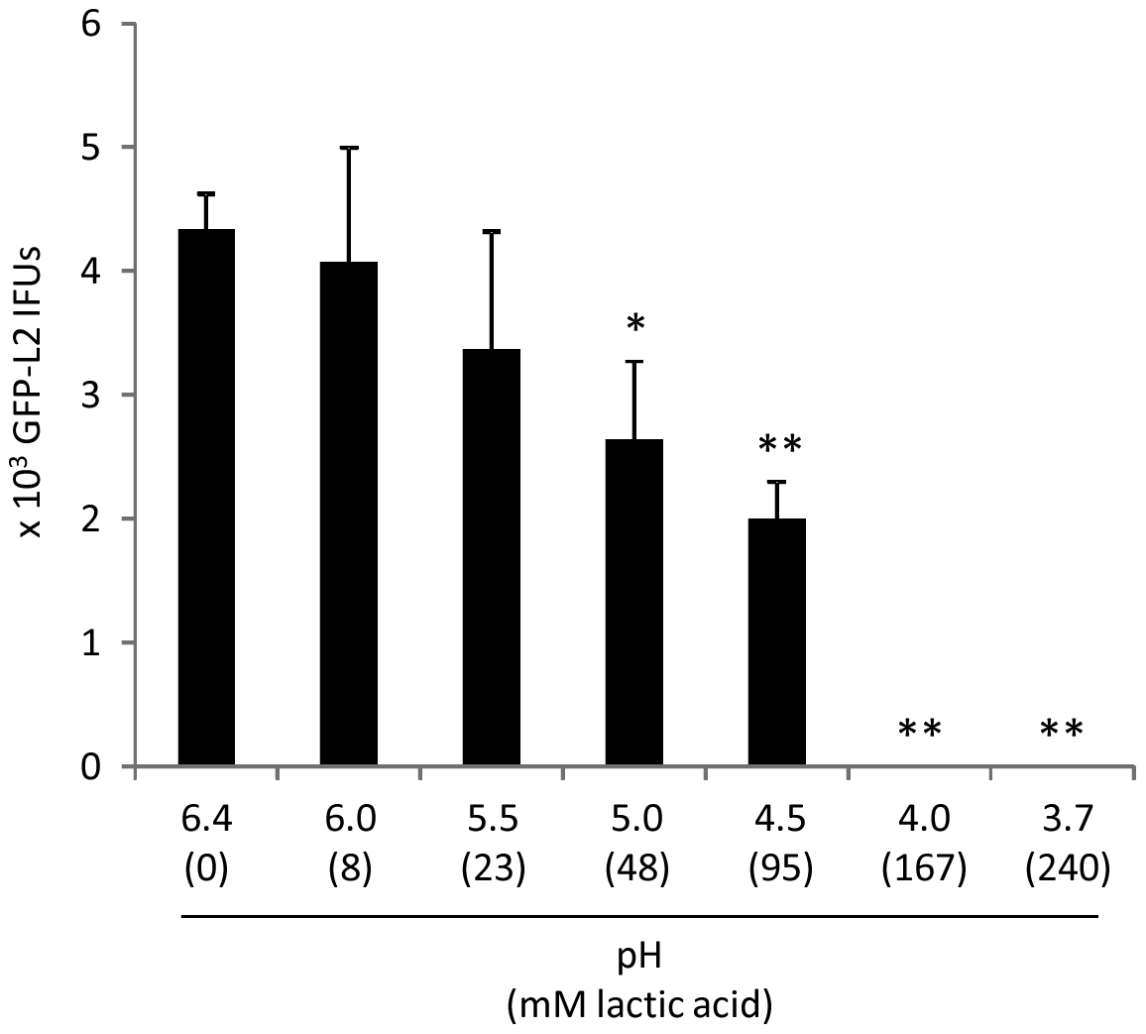
Killing of GFP-L2 EBs by lactate-acidified MRS. Experiments were performed similarly to those in Fig. 2 and 3; acidified MRS with indicated pH values were used in place of LCM. Values were averages ± standard deviations of triplicate experiments. Single and double asterisks above LCM-treated samples denote statistically decreased IFUs (P<0.05 and P<0.01, respectively) as compared to control pH-unadjusted MRS (pH 6.4). Note resemblance of inhibition trends exhibited by lactate-acidified MRS (this figure) and longitudinally harvested LCM with different pH (Fig. 2).

We next used lactic acid to adjust MRS to different pH values, and used the resulting media in place of LCM for chlamydial killing experiments. Lactic acid-acidified MRS exhibited an EB-killing trend (Fig. 4) that remarkably resembles the trend displayed by LCM with different pH values shown in Fig. 2. Taken together, data presented in Figs. 2–4 indicate that *Lactobacillus-*generated acidity is not only required but also sufficient for inactivation of L2 EBs.

### Lack of effects of catalase on chlamydiacidal activity of LCM

**Figure 5:**
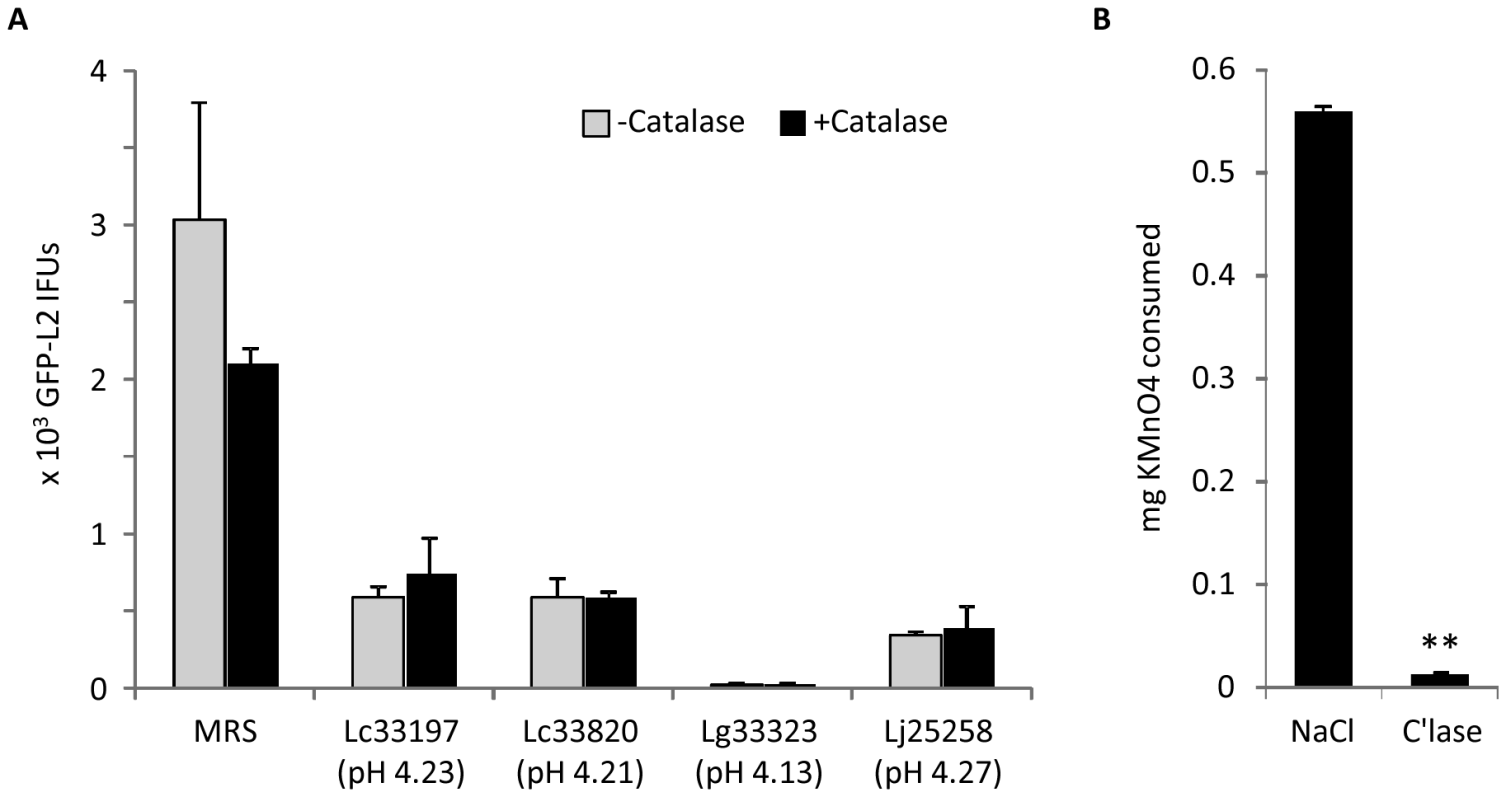
Lack of an effect of catalase on LCM-mediated chlamydiacidal activity. (A) LCM collected from still cultures of *Lactobacillus* strains were either untreated or treated with catalase before they were used to treat GFP-L2 EBs for 1 h. Surviving bacteria were quantified as outlined in Fig. 1 legend. Values were averages ± standard deviations of triplicate experiments. Note no statistical differences existed between catalase-treated and untreated samples for LCM from any of *Lactobacillus* strains. (B) 0.3% H_2_O_2_ prepared in 0.17 M lactate-NaOH (pH 4.0) was treated with 0.2 mg/ml catalase or control 0.9% NaCl. Remaining H_2_O_2_ was detected with KMnO_4_ titration. Values were averages ± standard deviations of triplicate experiments. Double asterisks signify statistically significant difference (P<0.01) in the amounts of H_2_O_2_ between catalase-treated and non-treated samples, and indicate that the catalase degraded H_2_O_2_ in the acidic LCM used in (A).

The highly similar, pH-dependent chlamydiacidal kinetics exhibited by LCM (Fig. 2) and lactic acid-supplemented MRS (Fig. 4), and the complete reversal of the killing by pH neutralization (Fig. 3) suggest that acid is primarily responsible for the chlamydiacidal effect of LCM since MRS does not contain any H_2_O_2_. We next assessed if pretreatment of LCM with catalase would weaken the antichlamydial activity. For these experiments, we used LCM with pH near 4.2. We reasoned that a role for H_2_O_2_ (if exists) in chlamydial killing should be detected following catalase treatment since LCM at pH 4.2 demonstrated only partial chlamydiacidal activity (Fig. 2A & Fig. 3). As shown in Fig. 5A, catalase pretreatment did not alter the efficacy of LCM collected from any of the tested *Lactobacillus* strains. To ascertain that the catalase is enzymatically active in such an acidic environment, we determined the amounts of KMnO_4_ needed to titrate H_2_O_2_ in catalase-treated and control saline-treated 0.3% H_2_O_2_ solutions prepared in 0.17 M lactate-NaOH (pH 4.0). Evidently, catalases effectively degraded H_2_O_2_ at pH 4.0 (Fig. 5B). These data further support that acidity is fully accountable for the chlamydiacidal activities in LCM.

### pH-dependent killing of GFP-CTD1 by LCM

**Figure 6:**
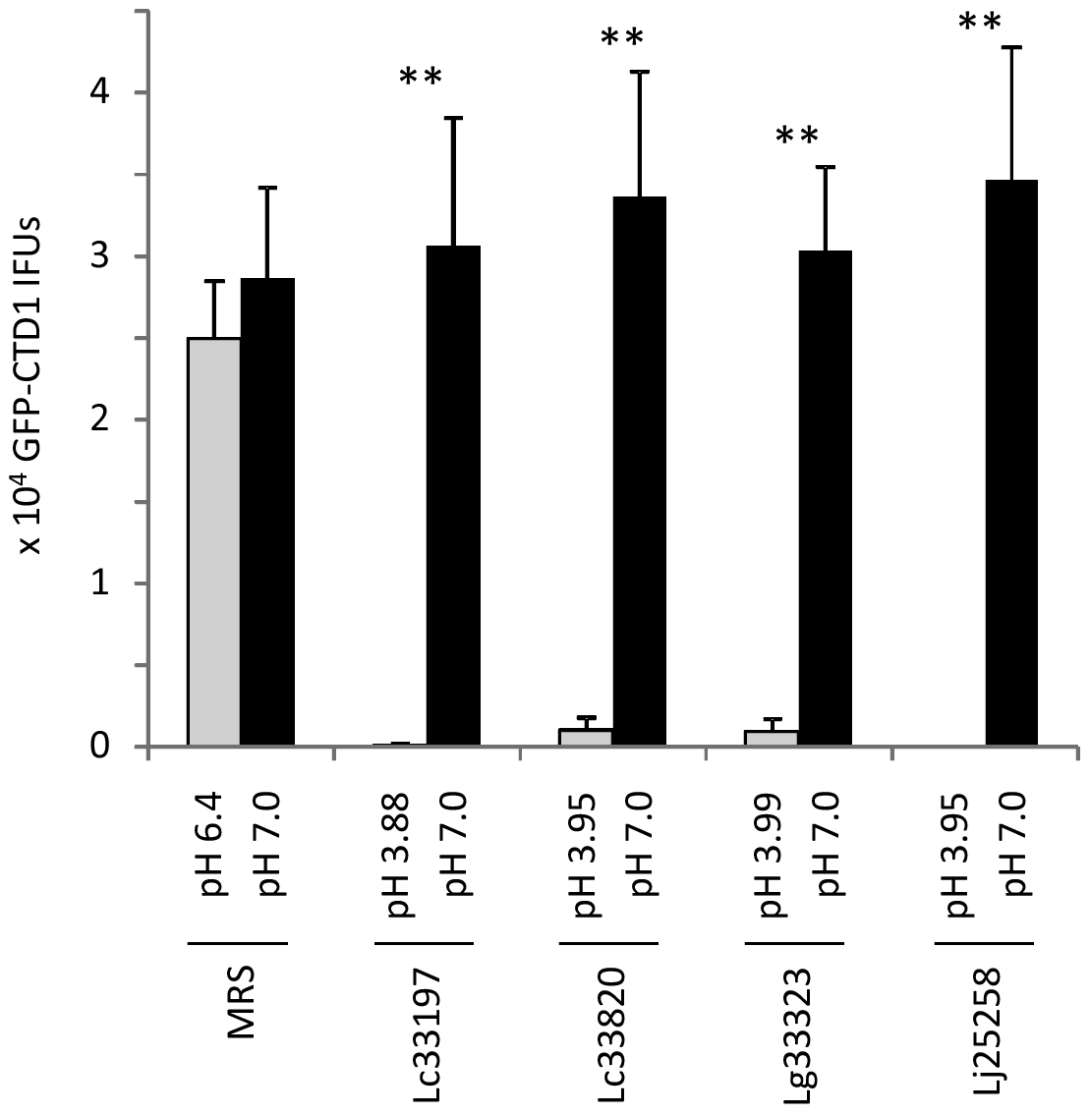
Acid-dependent inactivation of EBs of *C. trachomatis* serovar D-derived GFP-CTD1 by LCM. Experiments were carried in the same manner as those in Fig. 3 except centrifugation was used to facilitate infection. Values were averages ± standard deviations of triplicate experiments. Double asterisks indicate statistically significant difference (P<0.01, respectively) in the numbers of surviving EBs between treatment with LCM and that with neutralized LCM (pH 7.0).

All data presented above were obtained with *C. trachomatis* serovar L2 for the sake of experimental convenience. We then extended our study to GFP-CTD1, which was derived from an orthologous strain of *C. trachomatis* serovar D [34]. Similar to GFP-L2, the non-LGV genital strain was also highly susceptible to LCM prepared from still cultures of all *Lactobacillus* strains, and furthermore, neutralization of LCM with NaOH resulted in complete reversal of their chlamydiacidal activities (Fig. 6). These results suggest that *Lactobacillus-*generated acidity is fully responsible for inactivating *C. trachomatis* serovar D (and perhaps other non-LGV genital serovars) as well.

### H_2_O_2_ in *Lactobacillus* still cultures insufficient for *C. trachomatis* killing

**Figure 7:**
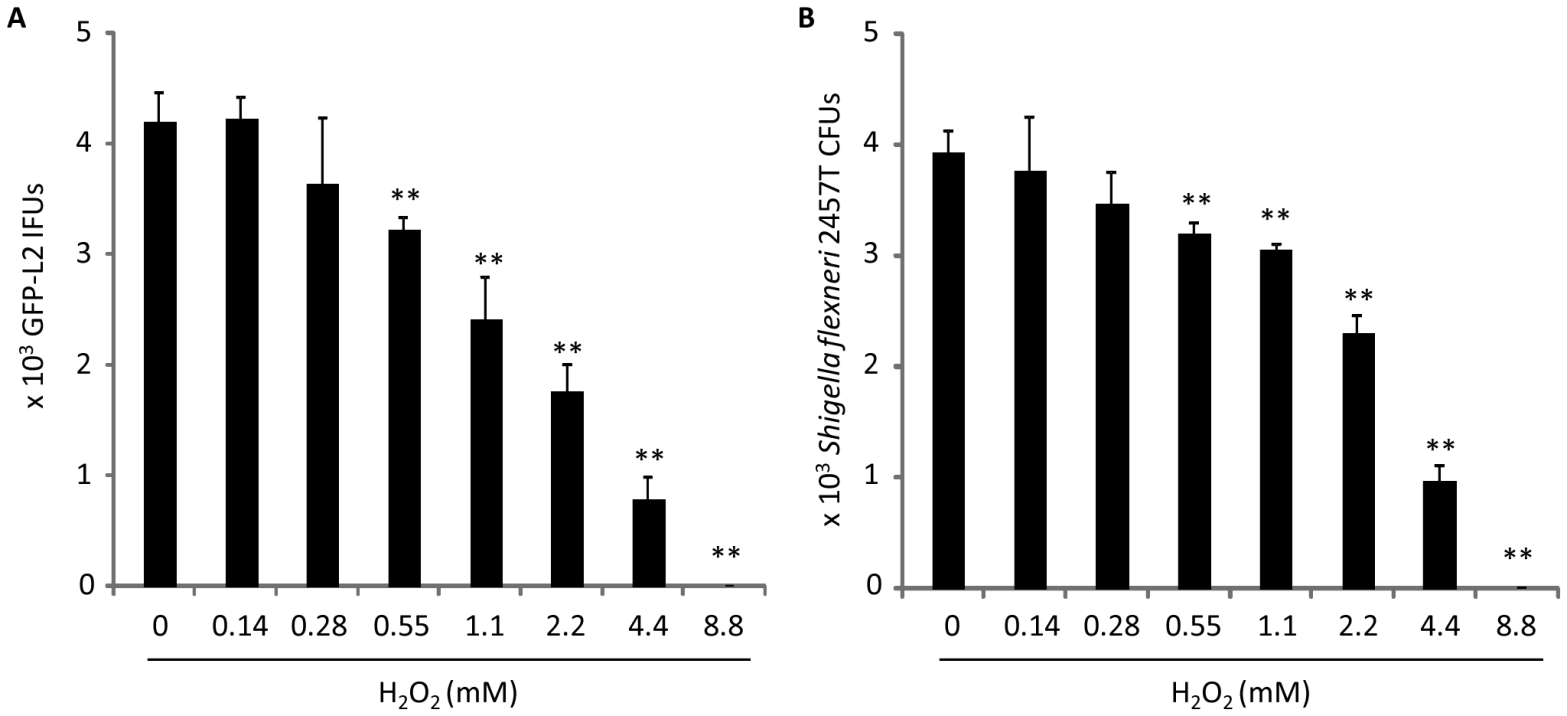
Dose-dependent killing of *Chlamydia* (A) and *Shigella flexneri* (B) by exogenous H_2_O_2_. GFP-L2 or *S. flexneri* 2457T was diluted in MRS containing indicated concentrations of H_2_O_2_. Following 1 h incubation at room temperature, remaining H_2_O_2_ was removed by catalase. Surviving EBs were quantitated as in Fig. 1; surviving 2457T bacteria were quantified by scoring colony-forming units (CFU) on LB Agar plates inoculated with serially diluted bacterial suspension. Values were averages ± standard deviations of triplicate experiments. Single and double asterisks signify statistically decreased live bacteria in MRS with an indicated concentration of H_2_O_2_, as compared to control non-supplemented MRS (P<0.05 and P<0.01, respectively).

An undetectable role for H_2_O_2_ in chlamydial killing by LCM could be because 1) H_2_O_2_ was not present or too low in our cultures even though previous studies have shown that all the *Lactobacillus* strains used in this study are H_2_O_2_ producers [37], [38], [39], 2) the spore-like chlamydial EBs are strongly resistant to H_2_O_2_, or 3) a combination of both. Using a highly sensitive commercial kit with a detection limit of 1 µM, the concentrations of H_2_O_2_ in the LCM from still cultures of Lc33197, Lc33820, Lg33323 and Lc25258 after overnight growth in a 5% CO2 incubator were measured to be 19.3±2.2, 34.2±3.5, 54.4±8.3 and 28.4±3.1 µM (averages ± standard deviations of triplicate experiments), respectively. Dose-effect analyses revealed that 8.8 mM H_2_O_2_ was the minimal concentration required to fully inactivate GFP-L2, whereas 0.55 mM was the minimal concentration that displayed a statistically significant killing effect (Fig. 7A). This minimal partially effective concentration was at least 10 fold higher than the H_2_O_2_ concentrations found in LCM, which explains why a role for H_2_O_2_ in LCM*-*mediated chlamydial killing was not detected in previous experiments.

In experiments with treatment procedures similarly to those used for GFP-L2, the free-living *Shigella flexneri* 2a 2457T demonstrated almost identical dose-dependent susceptibility to H_2_O_2_ (Fig. 7B), as compared with GFP-L2 (Fig. 7A). For *E. coli* BW25113 the minimal complete bactericidal concentration was above 8.8 mM (8.8 mM was the highest concentration tested), whereas the minimal partially effective concentration remained 0.55 mM (data not shown). Thus, compared with some other bacteria, *Chlamydia* did not appear to be particularly tolerant to H_2_O_2_.

### Shaking stimulates *Lactobacillus* H_2_O_2_ production, decreases lactic acid production and reduces chlamydiacidal activity

**Figure 8:**
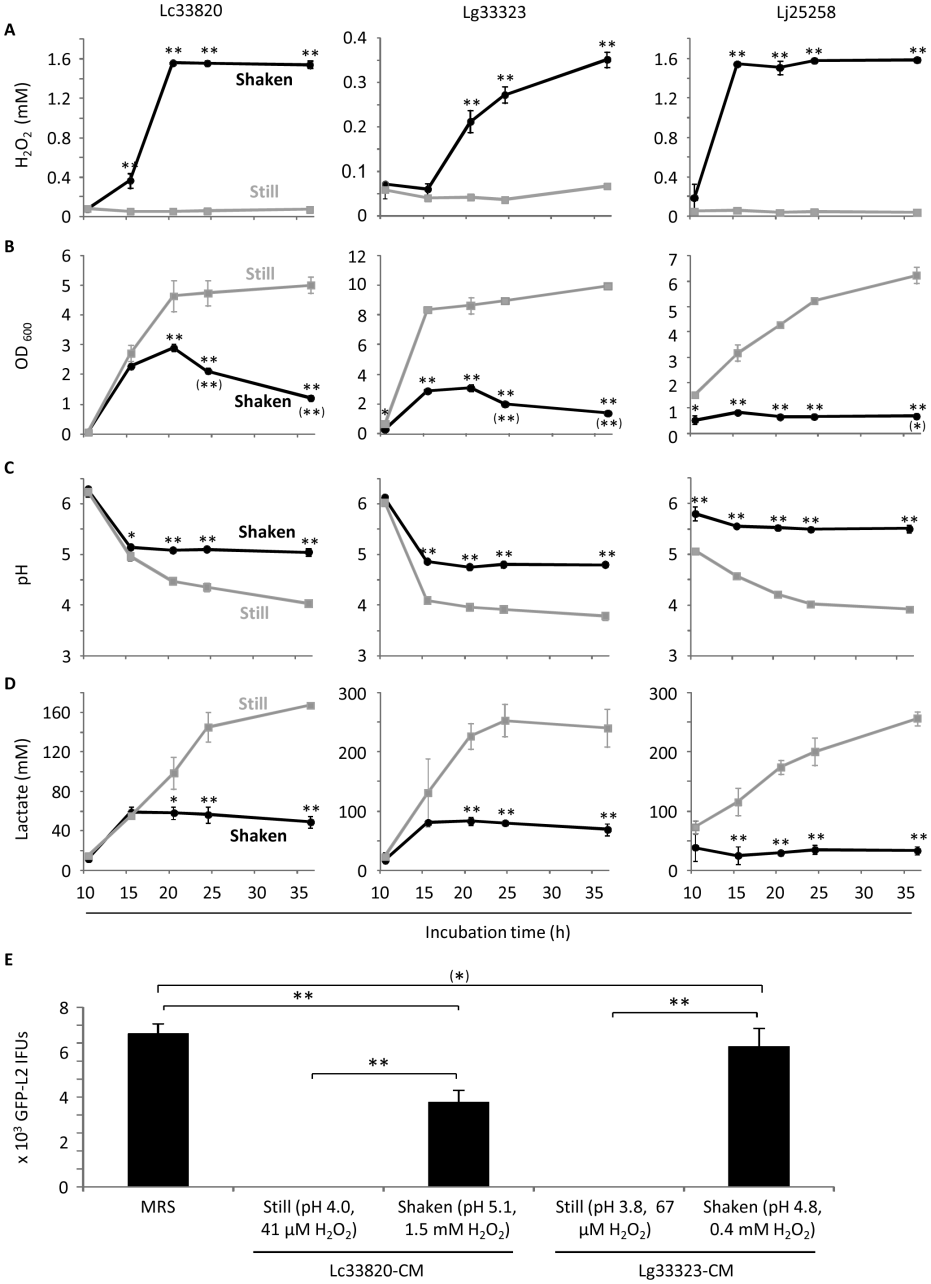
Induction of H_2_O_2_ production coupled with decreased lactic acid production and loss of chlamydiacidal activity. Overnight *Lactobacillus* cultures were diluted with fresh MRS to 0.02 OD_600_. The diluted bacterial suspensions were incubated as still cultures or shaken cultures as described in “Materials and Methods”. At indicated times, samples were taken for determination of the concentrations of H_2_O_2_ (A) and bacteria (B), pH values (C), lactic acid concentrations (D) and anti-chlamydial activities (E). Values were averages ± standard deviations of triplicate experiments. (A-D) Single and double asterisks indicate statistically difference (P<0.05 and P<0.01, respectively) between still cultures and shaken cultures at indicated times. Single and double asterisks in parentheses in B denote statistically significant decline in bacterial concentration following its peak at 20 h (Lc33820 and Lg33323) or 15 h (Lj25258). Double asterisks in E signify statistically significant differences (P<0.01) between indicated two groups. An asterisk in parenthesis indicates that the difference between control MRS and LCM from Lg33323 shaken cultures was deemed statistically insignificant by a two-tailed *t* test (P<0.1), but significant by a one-tailed *t* test (P<0.05).

Studies have shown that aeration can efficiently stimulate H_2_O_2_ production in some *Lactobacillus* strains [40], [41]. We found that this was also true for all four *Lactobacillus* strains employed in this study. Compared to still cultures, shaken cultures placed in the same incubator generated higher levels of H_2_O_2_ starting at 15 or 20 h after inoculation (Fig. 8A). For Lc33820 and Lj25258, there was 20–30 fold higher H_2_O_2_ in shaken cultures starting 15 h; for Lg33323, there was 4–6 fold higher H_2_O_2_ in shaken cultures starting 20 h (Fig. 8A). Although multiple time analyses were not performed for Lc33197, we detected a 26 fold higher level of H_2_O_2_ in an overnight shaken culture compared to a control overnight still culture (data not shown).

Interestingly, we observed poor bacterial growth in *Lactobacillus* shaken cultures of all three strains (Fig. 8B). Furthermore, OD_600_ values of shaken cultures declined significantly starting at different points after initial peaking at 15 h or 20 h, which suggests net losses of bacterial cells possibly due to H_2_O_2_-mediated cell lysis. In addition to growth inhibition and/or cell lysis, shaken LCM were significantly less acidic (Fig. 8C). The increased pH values in the shaken cultures were apparently due to decreased productions of lactic acid (Fig. 8D) but not the increased formation of H_2_O_2_ since addition of H_2_O_2_ to unconditioned MRS to a final concentration of 2 mM did not alter the pH (data not shown).

The concentrations of H_2_O_2_ in the shaken cultures of Lc33820 and Lj25258 remained around 1.5 mM after peaking at 15–20 h, and in the shaken cultures of Lg33323 remained below 0.4 mM at 36 h (Fig. 8A). As shown in Fig. 7A, at 2.2 mM, exogenously added H_2_O_2_ killed only ∼50% EBs, 0.55 mM was the minimal partially effective concentration. The levels of H_2_O_2_ and poor acidification in the shaken cultures (Fig. 7A, C, D) predicted that LCM from these cultures would kill EBs less efficiently than LCM from still cultures. This was proven to be the case using LCM obtained from still cultures and shaken cultures of Lc33820 and Lg33323 at 36 h (Fig. 8E). Taken together, these findings not only support the notion that low pH is critical for *C. trachomatis* killing by lactobacilli, but also suggest a conflict between H_2_O_2_ formation and lactic acid production in all three *Lactobacillus* species tested, and induction of H_2_O_2_ formation reduces the efficacy of lactobacilli in chlamydial killing through decreasing lactic acid production.

### Effective *C. trachomatis* killing by formic acid and acetic acid

**Figure 9:**
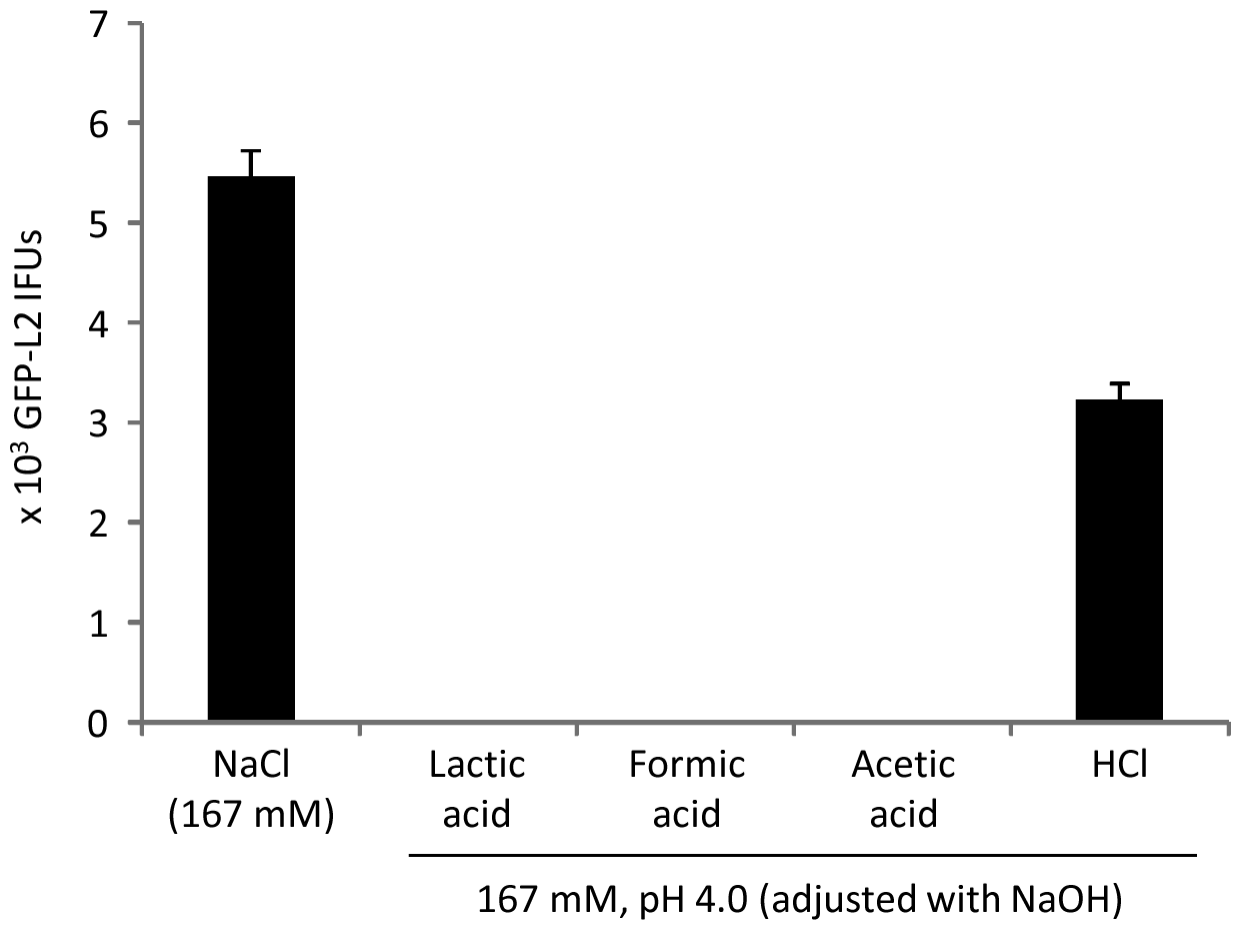
Full inactivation of GFP-L2 by formic acid and acetic acid but not pH-adjusted HCl. Experiments were carried in the same manner as those in Fig. 3 except acid solutions used to treat EBs were prepared in H_2_O.

All data presented above consistently suggests that low pH is essential for lactic acid-mediated chlamydial killing. We further accessed whether or not hydrogen ions released from compounds other than lactic acid could kill chlamydiae by determining the effects of formic acid, acetic acid and HCl on the viability of GFP-L2 EBs. Adjusted to pH 4.0 with NaOH, both a 167 mM formic acid solution and a 167 mM acetic acid solution inactivated all EBs (Fig. 9). However, 167 mM HCl (pH 4.0, adjusted with NaOH), which has a poor buffering capacity, inactivated only 40% of the EBs (Fig. 9). These results support the notion that a sufficient high concentration of hydrogen ions is critical for chlamydial killing by lactic acid or other acids.

## Discussion

In general, *Lactobacillus* species are considered probiotic microbes. While population-based studies have demonstrated a positive correlation between bacterial vaginosis, characterized by a lack of *Lactobacillus* species among other clinical features, and STIs including chlamydial STI [19], [20], [21], [22], [23], [24], [25], this report provides experimental evidence for inhibition of *C. trachomatis* by the probiotic bacteria. With sufficient acidity, LCM strongly inactivates both LGV and non-LGV genital *C. trachomatis*. Although for the sake of efficiency results presented in this paper were obtained using GFP-expressing *C. trachomatis* D and L2, we have found that wild-type organisms are equally susceptible to lactic acid (data not shown).

Lactobacilli generate two most important types of antimicrobials, lactic acid and H_2_O_2_ [42], [43]. To determine the potential roles for each of the antimicrobials in anti-chlamydial activity, we chose H_2_O_2_-producing strains [37], [38], [39] for this study. This report provides multiple lines of evidence consistently suggesting that low pH is fully responsible for the observed chlamydiacidal effect of lactobacilli. First, progressive chlamydiacidal activity was detected in LCM longitudinally collected from still cultures with progressively increased acidity (Fig. 2). Second, the chlamydiacidal kinetics exhibited by LCM obtained from still cultures (Fig. 2) and lactic acid-supplemented MRS (Fig. 4), which is free of H_2_O_2_, are strikingly similar. Third, neutralization of LCM resulted in complete reversal of chlamydiacidal activity (Fig. 3). Finally, poor antichlamydial activities were detected in LCM collected from shaken cultures with reduced lactic acid production (Fig. 8).

It has been very recently reported that the concentration of lactic acid in the cervicovaginal fluid in women ranges from 88–165 mM [44]. Significantly, in our experiments, 48–167 mM lactic acid in MRS can either partially or fully inactivate chlamydial EBs (Fig. 4). Therefore, both the pH values and the lactic acid concentrations that demonstrated to have protective effects in our *in vitro* experiments are achievable in the human vagina. This implies that lactic acid is capable of protection against chlamydial STI in women, which is consistent with findings of decreased risks of chlamydial STI in women with high vaginal lactobacilli levels and lower vaginal pH, as compared to women with bacterial vaginosis who have low numbers of lactobacilli and higher vaginal pH.

We speculate three nonexclusive mechanisms for lactic acid-mediated *C. trachomatis* killing. First, the acid may inactivate an EB surface molecule(s), which are critical for host cell attachment and/or entry. Second, lactic acid may disrupt the integrity of the outer membrane by reducing disulfides in the outer membrane complex thought to be critical for maintenance of EB's viability. Finally, hydrogen ions could enter the EB, disabling cellular metabolism essential for early chlamydial development.

Some free-living and/or facultative intracellular bacteria are equipped with acid resistance systems, enabling them to survive highly acidic environments, which can be as low as pH 2 [45]. In nonacidophiles, there are glutamate-, arginine-, and lysine-dependent acid resistance systems, which involve glutamate decarboxylase, arginine decarboxylase and lysine decarboxylase, respectively [45]. However, there is no evidence that *C. trachomatis* uses any acid resistance system to aid in its infection in the female genital tract. Whereas functional arginine decarboxylase activities are expressed by the respiratory pathogen *C. pneumoniae* and other species, which display tropisms for non-acidified organs, the activity of the enzyme is either weak or not present at all in genital *C. trachomatis* serovars D, F and L2 due to mutations [46], [47]. Similarly, a putative lysine decarboxylase gene is found in genomes of *C. psittaci* and *C. avium* [48], [49], but not that of *C. trachomatis* [50], [51].

In contrast to lactic acid, H_2_O_2_ is unlikely to play a significant role in protection against chlamydial STI because the physiological 23±5 µM H_2_O_2_ in the cervicovaginal fluid is far below the 0.55 mM minimal partially effective concentration (Fig. 7). It is somewhat surprising that *C. trachomatis* displays essentially the same level of susceptibility to H_2_O_2_ as *S. flexneri*, because the *C. trachomatis* genome does not contain any annotated catalase/hydrogen peroxidase genes [50], [51], whereas the *S. flexneri* genome encodes two different catalases/hydrogen peroxidases [52]. It is possible that the rigid outer membrane of the EB has a major role in keeping H_2_O_2_ from entering, and/or other chlamydial enzymes such as the thiol peroxidase have acquired the capacity to detoxify H_2_O_2_.

One reason for low levels of cervicovaginal H_2_O_2_ is the relatively hypoxic vaginal lumen that lactobacilli live in. Studies of others [40], [41] and our findings in Fig. 8 have shown that efficient induction of H_2_O_2_ production from lactobacilli *in vitro* requires vigorous agitation to increase aeration. It is plausible that sexual intercourse may improve aeration in the vaginal lumen, and therefore stimulate H_2_O_2_ production in lactobacilli. However, H_2_O_2_ produced during this period is expected to be efficiently inactivated by cervicovaginal fluid and semen [31], and consequently fails to protect against chlamydial STI. Similar to our study, previous reports have concluded that lactobacilli-derived H_2_O_2_ plays no practical roles in protection against herpes simplex virus type-2, *Neisseria gonorrhoeae*, *Hemophilus ducreyii* and bacteria associated with bacterial vaginosis [18], [31].

There have been considerable efforts to identify high H_2_O_2_-producing lactobacilli for probiotic use in women (eg., [22], [26], [27], [28], [29], [30], [37]). However, it is unlikely that efficient H_2_O_2_ producers identified *in vitro* are able to produce high levels of H_2_O_2_ in the relatively hypoxic vaginal lumen. Furthermore, efforts to increase vaginal aeration as a strategy to increase H_2_O_2_ production should be discouraged anyway since we have shown that induction of H_2_O_2_ generation leads to inhibition of lactic acid production and loss of chlamydiacidal activity.

There may be two mechanisms for the decrease in lactic acid production in highly aerated cultures. First, high concentrations of H_2_O_2_ may be toxic to producing cells since bacteria in shaken cultures fail to grow efficiently and/or are lysed (Fig. 8). Second, since a biochemical reaction that generates H_2_O_2_ in lactobacilli is coupled with pyruvate catabolism, induction of H_2_O_2_ formation stimulates the conversion of lactate to pyruvate [41]. Thus, efforts to maintain a sustainable acidity through regulating lactic acid production and preservation are more sensible than those to upregulate H_2_O_2_ formation. Significantly, in a randomized, double-blind and placebo-controlled trial among patients with bacterial vaginosis, a single dose of tinidazole and lactobacilli resulted in decreased vaginal pH and significantly improved cure of the disease, compared to a single dose of tinidazole in combination with placebo [32]. Hopefully, lactobacilli that efficiently produce lactic acid can be used for prevention of STI caused by *C. trachomatis* and other pathogens.

Development of effective topical microbicides against STI pathogens is an area that has been actively explored by researchers for some time. The fact that formic acid and acetic acid effectively inactivated EBs suggest that other weak acids in addition to lactic acid, in principle, may be incorporated into topical microbicides, provided that those acids do not adversely affect cells in the genital tract or lactobacilli. However, evidence suggests that acetic acid, in reality, might be of limited value as a vaginal microbicide component. First, its relatively high p*K*a value (4.76) makes it less effective than lactic acid, which has a p*K*a value of 3.86, in acidification of the vaginal environment to pH 4.0 or lower for efficient inactivation of chlamydiae (and other STI pathogens). Consistent with this reasoning is our observation that adjustment of MRS to pH 4.0 requires 453 mM acetic acid, compared to 167 mM lactic acid. It has also been reported when a 1% acetic acid solution and a 1% lactic acid solution were both adjusted to pH 3.8, the acetic acid solution inactivated HIV less efficiently, even though the molar concentration of acetic acid was higher than that of lactic acid [53]. Although we have not compared the chlamydiacidal activities of lactic acid and acetic acid at concentrations lower than 167 mM, with the aforementioned information we suspect that kinetic analyses would reveal less efficient *C. trachomatis killing* by acetic acid as well.

In contrast to acetic acid, potential utility of formic acid, whose p*K*a value (3.77) is very close to that of lactic acid (3.86), as a microbicide candidate, warrants consideration. Formic acid has been tested as an antibiotic replacement in poultry feeds [54], [55]. Whereas the acid minimized infection by *Salmonella* from experimentally contaminated food [54], it also reduced the number of lactic acid-producing bacteria in the crop of broilers [55]. Therefore, if formic acid is explored as a microbicide component for STI prevention, how the acid may influence human vaginal *Lactobacillus* species needs to be examined very carefully.

In addition to lactic acid and H_2_O_2_, lactobacilli also produce a large group of antimicrobial peptides [16], [17]. There is no evidence that antimicrobial peptides play a significant role in LCM-mediated chlamydiacidal activity. However, since we have not measured antimicrobial peptides in LCM, we cannot exclude the possibility that they contribute to the antichlamydial activity of lactobacilli *in vivo*.

During the time that this work was reviewed for publication, Mastromarino *et al.* reported moderate inhibitory effects of *Lactobacilli brevis* and *L. salivarius* on chlamydial infection in cell culture [56]. In their report, modest reductions of IFUs were observed after mixing *C. trachomatis* organisms with high numbers of lactobacilli in PBS or by incorporation of lactobacilli into cell culture during the attachment/entry period. Their findings, together with our data reported here, suggest that lactobacilli may compete with host cells for binding incoming chlamydiae that are not killed due to insufficient acidity in the vagina [56]. In addition, they also observed decreased chlamydial growth from infected cells cultured in the presence of lactobacilli [56]. This phenomenon could be consequent of activation of the innate defense system in the vaginal epithelia by the probiotic bacteria [57], [58] although it is also possible that the presence of lactobacilli reduced nutrients for infected cells. Whereas lactobacilli may inhibit chlamydial infection through multiple mechanisms, lactic acid-mediated EB killing is clearly the most efficient one.

In summary, we have shown that lactic acid but not H_2_O_2_ is both required and sufficient for the antichlamydial activity of three *Lactobacillus* species that dominate the human vaginal microbiome. Stimulation of H_2_O_2_ production in lactobacilli leads to inhibited *Lactobacillus* growth, decreased lactic acid production, and loss of antichlamydial activity. These findings have important implications for development of lactobacilli as prophylactic and therapeutic agents for chlamydial STI and other infectious diseases in the genital tract.
